# Enhancing Organizational Memory Through Virtual Memoryscapes: Does It Work?

**DOI:** 10.3389/fpsyg.2021.683870

**Published:** 2021-08-12

**Authors:** Antonio Mastrogiorgio, Francesca Zaninotto, Francesca Maggi, Emiliano Ricciardi, Nicola Lattanzi, Andrea P. Malizia

**Affiliations:** ^1^AXES, IMT School for Advanced Studies Lucca, Lucca, Italy; ^2^MoMiLab, IMT School for Advanced Studies Lucca, Lucca, Italy; ^3^Intesa Sanpaolo Innovation Center S.p.A., Turin, Italy

**Keywords:** virtual reality, memory offloading, digital amnesia, organizational memory, virtual memoryscapes

## Abstract

Enhancing cognitive memory through virtual reality represents an issue, that has never been investigated in organizational settings. Here, we compared a virtual memoryscape (treatment) – an immersive virtual environment used by subjects as a shared memory tool based on spatial navigation – with respect to the traditional individual-specific mnemonic tool based on the “method of loci” (control). A memory task characterized by high ecological validity was administered to 82 subjects employed by large banking group. Memory recall was measured, for both groups, immediately after the task (Phase 1) and one week later (Phase 2). Results show that (i) in Phase 1, the method of loci was more efficient in terms of recalling information than the to the virtual memoryscape; (ii) in Phase 2, there was no difference. Compared to the method of loci, the virtual memoryscape presents the advantages – relevant for organizations – of being collective, controllable, dynamic, and non-manipulable.

## Introduction

With the rise of information technology and digitalization, memory offloading is becoming paramount in organizations. Information systems represent devices in which knowledge can be externalized to be stored, shared, and easily accessed to support decision-making (Stein, 1995; Alavi and Leidner, 2001; Barros et al., 2015). However, this massive memory offloading onto artificial devices comes at a price. The more we are used to offloading information, the less we rely on our memory (we quickly realize that the more pictures we take with our phone, the less we need to remember the things we see). There is evidence – also known as “*digital amnesia*” or “Google effect” – of a generalized decrease of memory performance: rates of recall of specific information depends on how much subjects expect to rely on external sources, such as the information on the Internet (Sparrow et al., 2011; Wegner and Ward, 2013). As discussed by Sparrow et al. (2011, p. 776): “The Internet has become a primary form of external or transactive memory, where information is stored collectively outside ourselves.” Digital amnesia is critical in organizations in which not just the Internet but also dedicated intranets are heavily used to support decision-making. The more the information technology allows offloading a significant amount of fine-grained information on external devices, the less organizational actors rely on their memory (for a discussion, see Heersmink, 2016). Despite the advantage of freeing cognitive resources, offloading is often maladaptive and impairs memory performance (Carr, 2011; Greenfield, 2014).

How to enhance the cognitive memory of organizational actors is a relevant topic that is gaining in importance in the age of digital amnesia, acknowledging that not everything can be offloaded. In many organizational contexts, information must be strictly memorized to be cognitively handled and effectively used. While the well-known “transactive memory theory” (Wegner et al., 1985; Wegner, 1987; Bachrach et al., 2019) assumes that different subjects of a social group possess different information – memory is distributed – how to align information among individuals – *memory is shared* – is a relevant matter, unaddressed in current research. The problem significantly characterizes teamwork. We easily realize that some information is externalized onto dedicated systems, while other is distributed among team members.

Nevertheless, there is another type of information that cannot be offloaded on external artifacts, neither distributed among team members. Indeed, there are types of information that are simply shared: all the team members must be aligned; that is to say, *they all need to retain and recall the same information*. In addition, often, such information creates a significant cognitive burden, is continuously updated, and must be retained without errors (for the sake of simplicity, think about a team working on the design of a novel complex product so as all team members need to retain and quickly recall the general layout of the product in order to accomplish their tasks).

In this contribution, we argue that the current emphasis on information systems, as offloading devices, is diverting the attention of scholars from a number of alternative, cutting-edge applications of digital technologies. Such applications could be relevant to enhance organizational memory. *De facto*, digital technologies allow creating digital memoryscapes, that can be used by organizational actors as collective memory tools. While the non-technical notion of memoryscape refers to spatial representations of socially shared memory (Butler, 2007; Kappler, 2017), a *virtual memoryscape* is an immersive virtual environment used by subjects as a shared memory tool based on spatial navigation (cfr., Krokos et al., 2019). A virtual memoryscape, being – by definition – non-individual, can be used to create, in a controllable manner, a memory shared among the members (for a discussion on collective memory, see Hirst and Manier, 2008).

The use of virtual reality to enhance memory is also an emerging method in neuroscience research (Mishra et al., 2016; Wais et al., 2021). Evidence shows that memory processes involve spatial navigation, that is to say, superior memorizers tend to associate specific information to specific places, requiring a visual-motor experience (Maguire et al., 2003; Buzsáki and Moser, 2013; Hartley et al., 2014). The well-known mnemonic strategy called “Method of Loci,” hereafter MoL (Yates, 1966; Legge et al., 2012, where “locus/loci” is the Latin of “place/places”) requires associating the items to be remembered to specific experienced places. The involvement of active visual-motor interaction with the environment by leveraging vestibular and proprioceptive senses is functional to memorization (Brooks, 1999). Virtual reality allows creating “memorable” experiences to enhance productivity through a better recall of information (Krokos et al., 2019). The use of virtual reality as a collective memory tool represents a cutting-edge application in organizational settings and represents a tool to counterbalance the maladaptive consequences of massive offloading to “fix” digital amnesia in organizations.

In our study, we investigate the efficacy of a virtual memoryscape (where the locus is identical for all the subjects and is implemented through virtual reality) compared to traditional MoL (where the locus is individual-specific and mentally represented). We administered a memory task characterized by high ecological validity to 82 subjects with responsibilities in a business unit of a large banking group. Memory performance measured as memory recall was compared between a treatment group that used the virtual memoryscape and a control group that used the traditional MoL. The results highlight that, in the long-term, the virtual memoryscape and the traditional MoL are equivalent. The results are relevant as the virtual memoryscape presents a number of advantages with respect to the traditional MoL. Indeed, the virtual memoryscapes is (a) collective (the memoryscape is shared among organizational actors); (b) controllable (the memoryscape can be managed by a central authority); (c) dynamic (the memoryscape can be easily updated); and (d) non-manipulable (the memoryscape cannot be easily adulterated), as compared to individual usage of MoL.

### Mnemonics

The “method of loci” (MoL) is the oldest known mnemonic strategy used to recall a sequence of items. It consists of imaging to place the items in specific locations (“loci” is the Latin for “places”) of a familiar environment so that a person will be able to recall the items simply by visualizing the environment (Yates, 1966). MoL has been used since ancient Roman and Greek times and is also called the “memory journey” or “memory palace” because familiar places, such a house or a palace, were used as “loci”: items to be remembered were ideally placed and recalled by mentally walking through the familiar environment. MoL is surprisingly effective as it allows memorizing a relevant number of items in a relatively easy manner. It is used in memory competitions, in which skilled individuals have to memorize the largest number of items (Raz et al., 2009). While memory champions can memorize a hundred items in a matter of minutes, unskilled individuals are able to memorize dozens of items even if they are applying the method for the first time. This makes MoL surprisingly powerful as it is relatively effortless considering its immediate results. Learning to link items to a place is a relatively easy task that can radically improve declarative memory with a relatively limited training time (Legge et al., 2012). The neural substrates involved in the MoL are “self-explaining”: superior memorizers do not possess exceptional intellectual abilities or abnormal brain structures, but they are simply proficient in applying spatial navigation, based on the hippocampus, as a memory tool (Maguire et al., 2003; see also Mallow et al., 2015). Brain areas involved in spatial navigation and memory are connected: how we move in and perceive the environment influences the way we create our memory, that is to say, how we retain and recall contents (Buzsáki and Moser, 2013; Hartley et al., 2014).

However, the cognitive pillar of MoL also constitutes its main limitation: the familiar environment, in which items are ideally placed to be recalled, cannot be controlled across individuals. MoL relies on idiosyncratic resources, as different individuals use different places (i.e., everyone uses their own house) as a memory tool. This limitation is probably why MoL has been mainly used and investigated with reference to single individuals, while applications to larger social groups, such as the whole organization, are quite marginal. In other words, because loci are individual-specific, they could involve variables that cannot be controlled. In order to overcome these difficulties in experimental research, an experimenter-supplied environment, identical for all the participants, has been used (Jamieson and Schimpf, 1980; Moè and De Beni, 2005).

### Virtual Reality and Memory

Virtual environments represent a substantial innovation for the application of MoL, as they allow creating “virtual palaces” that present the significant advantage of being immersive: such palaces do not need to be mentally represented, but they are implemented through a computer interface. The use of virtual environments presents some evidence, though limited (for an overview, see Krokos et al., 2019). Traditional MoL was compared with virtual environments, administered through a computer monitor (desktop condition). While some studies show that virtual environments are superior to traditional MoL (Legge et al., 2012), others show no difference between conditions (Fassbender and Heiden, 2006). What makes the difference is not the use of virtual environments *per se*, but that the virtual environments are *immersive* and can be experienced as 3D realistic spaces. Evidence of better memory recall is related to added dimensionality, such as using more displays to create a visual angle (Bowman and McMahan, 2007; Ragan et al., 2010). Indeed, there is solid evidence that visual- and motor-related processes influence memory (Maguire et al., 2003; Madan and Singhal, 2012a,b). Considering that the hippocampus plays a central role in both long-term memory and spatial navigation (e.g., Rolls and Xiang, 2006), it is possible to enhance memory (also for therapeutic interventions) through the experience of enriched environments administered through videogames and virtual reality (Clemenson and Stark, 2015; Mishra et al., 2016; Wais et al., 2021).

A fundamental and relatively recent innovation relies on the possibility of implementing MoL in virtual reality, in particular using a head-mounted display (hereafter HMD) which provides a stereoscopic field of regard, through distinct images for each eye, to allow a 3D realistic immersion into the environment (Shibata, 2002; Bowman and McMahan, 2007). As discussed by Krokos et al. (2019, p. 1): “HMD condition provide a superior memory recall ability compared to the desktop condition (2D). We believe this is a first step in using virtual environments for creating more memorable experiences that enhance productivity through a better recall of large amounts of information organized using the idea of virtual memory palaces.” Virtual reality represents a fresh and novel application for memory recall: it allows creating virtual “memory palaces,” that, involving a significant visual-motor experience, provide a strong association between the content to be retained and its spatial placement. Virtual reality is connoted by the fact that the individual perceives to be included in and interacting within an environment (Steuer, 1992; Slater et al., 2010). In particular, when place illusion (the sensation of “being there” in a real place) and plausibility illusion (the illusion that the scenario being depicted is actually occurring) occur, participants respond realistically to the virtual reality (Slater, 2009).

The possibility of an active visual-motor interaction with the environment, by leveraging vestibular and proprioceptive senses instead of a passive visualization, enhances memory and increases recall rates (Brooks, 1999). The use of virtual reality, implemented through an HMD, has been analyzed with reference to navigation time (Ruddle et al., 1999) and cognitive awareness of objects (Mania et al., 2003) and compared with a control group represented by desktop condition (where the spatial navigation was implemented through a computer screen). Vindenes et al. (2018) implemented MoL in a virtual environment, demonstrating that subjects with higher spatial reasoning abilities benefit more from the use of the MOL. Virtual reality was used to promote transfer: memory training to older adults with memory impairment increased performance on virtual reality memory tasks (Boller et al., 2021), in contrast with previous evidence showing that playing brain-training games in virtual environments did not improve the transfer of cognitive training (Parong and Mayer, 2020). A fundamental aspect of learning in a virtual environment pertains to the role of haptics (which is crucial in specific settings such as medical simulators, see Coles et al., 2010). Morimoto (2020) shows that haptic working memory and visual working memory share a common storage system. Memorization and recall are processed by different brain areas as there is a significant difference in the integration of multisensory information between the exploration of an object for encoding or the exploration for recalling purposes (Sciutti et al., 2019). Furthermore, social interaction also plays a role as immersion mediates person-virtual environment interaction effects on satisfaction and loyalty of VR applications (Hudson et al., 2019), contrary to previous findings showing that social interactions decrease the impact.

Very importantly, the widespread implementation of VR applications through HMD induces a number of symptoms and effects, so-called VRISE (Kourtesis et al., 2020) such as nausea, dizziness, disorientation, fatigue, and instability. Researchers’ technological competency on HMD hardware, software, and procedures is paramount to ensure the health and safety standards to minimize cybersickness symptomatology (Kourtesis et al., 2019), particularly when HMD is use for long periods.

### Research Gaps

Virtual reality, involving perception and action mechanisms, represents a relatively novel tool to enhance memory through spatial navigation in known architectures and places. The use of virtual reality as a tool for memory enhancement has never been investigated in organizational settings. Indeed, while previous studies (discussed in previous sections) have been conducted in non-ecological settings on non-representative populations (in particular, students, or patients), our study represents a novelty.

Virtual reality represents a new-generation tool for memory that creates virtual “memory palaces,” which can be easily exploited by larger social groups (i.e., teams) to enhance memory. In other words, virtual reality allows extending the benefit of the MoL to larger social groups, as it provides a shared memoryscape, where *the same conditions for spatial navigation apply to all the users*. The non-technical notion of memoryscape refers to such places and landscapes used to recall information of cultural importance (Butler, 2007; Kappler, 2017) and is related to the general consideration that memory, in its cultural meaning, is not just “a property of time” but it normally refers to the spatial presence in specific places. In our contribution, we extend the general notion of memoryscape to virtual memoryscape, and we define, for instrumental purposes, *virtual memoryscapes* (hereafter VM) as virtual environments used by organizational actors as a shared memory tool based on spatial navigation. The traditional distinction between individual memory and organizational memory (assumed to be an individual-like construct, Walsh and Ungson, 1991) takes on particular importance. While transactive memory theory (Wegner et al., 1985; Wegner, 1987; Bachrach et al., 2019) assumes that different subjects of a social group possess different information (information is distributed: specific actors possess specific information), how to align information among subjects (information is shared: all actors must retain and recall the same information) is a relevant problem in organizational settings, for which virtual reality comes in help.

As we explain in the next sections, our study was implemented in a real organization and is connoted by stringent ecological conditions: (i) participants were part of a large banking group in which they have responsibilities in a business unit; (ii) experimental task was like real tasks they accomplish in their standard working activities; (iii) spatial navigation occurred in virtual places consistent with real workplaces, experienced by the participants.

### Hypotheses

In our study, we investigate the efficacy of a virtual memoryscape (where the locus is identical for all the subjects and is implemented through virtual reality) in comparison with traditional MoL (where the locus is individual-specific and mentally represented).

We expect – *Hypothesis 1* – that *the traditional MoL is more effective than a VM in the short term. De facto* individual-specific familiar places (loci) are, from the cognitive point of view, more vivid and accessible, with respect to a novel virtual environment, so they constitute a more reliable short-term-memory tool (cfr., Jamieson and Schimpf, 1980; Moè and De Beni, 2005).

Short-term effects are trivial in organizational settings in which memory processes become critical when they involve long-term constructs. Then, we expect – *Hypothesis 2* – that *in the long term, a VM constitutes an equally, if not more, reliable memory tool as compared to traditional MoL, as it presents lower memory decay*. The potential reasons for hypothesizing a lower decay of memory after experiencing a VM is strictly related to the peculiarities of virtual reality (discussed in previous sections): virtual reality (used in the experimental group), allowing 3D immersive experience, exerts powerful cognitive imprinting, more effective than just “thinking about” familiar places (control group).

## Materials and Methods

### Participants

Eighty-two participants were recruited from a large banking group. They had responsibilities in the business unit of customer support and sales. Participants had a normal or a corrected-to-normal vision, no history of auditory or psychiatric disorders. The study was compliant with the ethical principles of the Declaration of Helsinki (World Medical Association, 2013) and was conducted under a protocol approved by the Area Vasta Nord Ovest Ethics Committee (protocol n. 24579/2018). Participants were provided with an exhaustive description of all the procedures and were required to sign a written informed consent. The average age of participants was 44.52 (St. Dev. = 7.67), 34% were females, and 66% were males.

### Task

Subjects involved in the experiment normally handle complex insurance contracts. Hence, we used the information of a (new but not publicly launched) insurance contract unknown to the subjects who participated in the experiment. We used as task an insurance contract to be remembered. Importantly, an insurance contract represents typical information that must be cognitively processed to be effectively handled. Such information can be undoubtedly offloaded onto external devices, but this offloading is somehow trivial, as this type of information must be cognitively retrieved by the decision-makers to be effectively used.

The use of a real insurance contract allows reproducing the ecological conditions normally faced by participants in their standard working activities. The memory task consisted of remembering specific contents of the new insurance contract. Such contents were represented by a list of words and descriptions, characterized by both numerical and textual information.

#### Phase 1

We created two randomized groups. We administered the memory task to the control group (*N* = 41) to be accomplished through the traditional MoL. First, MoL was explained to participants through the reading of descriptive information, then the contents of the insurance contract were listed in a textual manner on a screen. Participants applied MoL using individual-specific places (such as their own house) as memory tools. The memorization phase took 10 min, and then we measured the memory performance of participants through a 17-item questionnaire in which we asked them to recall specific information – words and descriptions – about the contract. We codified answers through a 5-point scale: from 0 (“totally incorrect”), through 0.5, 1, 1.5, to 2 (“totally correct”), so as to calculate a memory score for each participant (as the sum of items scores), where the maximum potential score was 34, resulting from 2 (maximum score for a “totally correct” response) × 17 (number of items).

**FIGURE 1:**
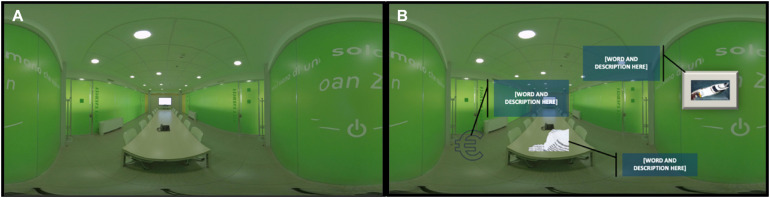
2D representation of the Meeting Room of the virtual memoryscape **(A)** with the objects and their pop-up windows in which words and descriptions were placed **(B)**.

We administered the memory task to the experimental group (*N* = 41) to be accomplished through the VM. The experimental group (*N* = 41) experienced the virtual memoryscape through an HMD (Oculus Go^TM^), allowing a 3D realistic immersion into the environment. The virtual environment had been specifically recorded by a 360-camera to resemble the real working setting experienced during everyday activities (same type of furniture and artifacts, similar layouts, etc.). This specific design of the virtual environment improves the ecological validity and is functional to the purpose of our study, implemented in strict organizational settings. The virtual memoryscape, see Figure 1A, was composed of five rooms: (1) Entrance, (2) Computer Room, (3) Meeting Room, (4) Book Crossing, (5) Break Room.

After a preliminary exploration session (10 min), the method of loci was explained through a text superimposed on the digital setting, and then participants entered again (for 10 min) into the virtual environment in which different objects were presented in each room. As shown in Figure 1B, by clicking each object, a pop-up window containing the word and the description was shown. Such words and definitions were the contents to be remembered, associated with different objects present in the virtual environment.

Immediately after the memory session, we measured the memory performance of participants through the 17-items questionnaire (described previously), in which we asked them to recall the specific information. The items were codified using a 5-point scale (from “totally incorrect” to “totally correct”). We calculated a memory score for each participant as the sum of the item scores.

#### Phase 2

In Phase 1, we assessed memory immediately after the memorization session. There are reasons to think that such memory performance was significantly based on the recency effect, which is the tendency to better recall the items at the beginning and the end of the administered list (Mack et al., 2017). Therefore, we cannot exclude that memory performance was significantly based on the information present in working memory, as the questionnaire was administered immediately after the use of MoL. Considering that our interest is not in working memory but in demonstrating the long-term efficacy of virtual memoryscapes in organizational settings, we replicated the 17-items questionnaire after a week, during which the participants had no opportunity to refresh the information to be memorized, in order to (re)assess the memory performance. Using the same questionnaire and same scoring system of Phase 1, we calculated the memory score for each participant after a week (Phase 2).

### Statistical Analysis

The memory scores in Phase 1 and Phase 2 can be considered as a proxy of a memory decay to be intended as a delta of memory performances. This memory decay is related to the opportunity of assessing long-term effects ruling out short-term effects, relying on the evidence that memory decay is due to time passage or interference of other memoranda (Berman et al., 2009). Notice that memory decay, in our experiment, is related to a time-window of a week, but the results related to a week can be likely, generalized to longer terms as we can reasonably assume that the decay is monotonic according to the well-known Ebbinghaus’ forgetting curve (Ebbinghaus, 1880), recently replicated (Murre and Dros, 2015).

We compared memory decay (between Phase 1 and Phase 2) related to treatment and control (VM and MoL, respectively). We first calculated descriptive statistics, then in order to test Hypothesis 1 and Hypothesis 2, we run a *two-factor ANOVA with repeated measures on one factor* after checking for the assumptions. Through the ANOVA, we compared treatment and control, calculating memory score in two different Phases for each subject (in which age and gender were covariates), along with effect size (using Hedges’ g and ω^2^, see Lakens, 2013). After that, we run a *post hoc analysis* for pairwise comparisons between control and treatment, in Phase 1 and Phases 2, using a *t-test* (evaluated through the Bonferroni adjustment). Statistical analyses were conducted with JASP (Version 0.14.1; JASP Team, 2020).

## Results

### Descriptive Statistics

Table 1 shows the descriptive statistics of memory recall scores for control (MoL) and Treatment (VM) measured in Phase 1 (immediately after the task) and in Phase 2 (one week later). While control group memory is characterized by a decrease in memory recall from 12.207 in Phase 1 to 8.256 in Phase 2, the experimental group memory performance seems to be stable, ranging from 9.561 in Phase 1 to 9.585 in Phase 2. The control group presents less variability (i.e., lower standard deviation) in both Phases than the experimental group. Pearson’s correlation between memory scores of the same individual in both Phases is negative: in particular, the moderate negative correlation in the control group (MoL) is significant and highlights that subjects with higher performance in Phase 1 present lower performance in Phases 2, suggesting a worsening in memory recall. The weak negative correlation in the experimental group (VM) is not significant.

**TABLE 1:** Descriptive statistics of memory recall.

Groups	Repeated measures	*Mean*	*St. Dev.*	*Pearson’s r*
MoL (control)	Phase 1	12.207	3.860	−0.342*
	Phase 2	8.256	3.659	
VM (treatment)	Phase 1	9.561	4.673	−0.038
	Phase 2	9.585	4.005	

Score range: from 0 to 34. *P minor equal 0.05.

**FIGURE 2:**
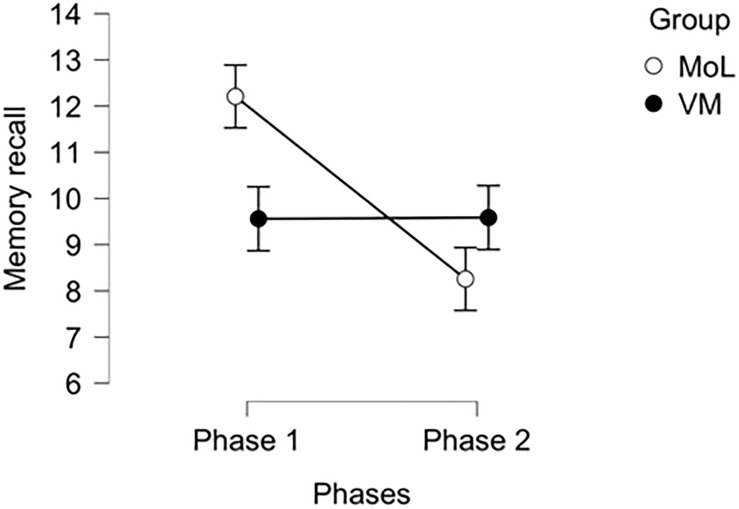
Memory recall for control (MoL) and treatment (VM) in Phase 1 and Phase 2.

Figure 2 shows memory scores in the previous table (37 is the maximum potential score, as described in section “Phase 1”). While for traditional MoL (control), memory recall drastically decreases between Phase 1 and Phase 2, for VM (treatment), there is no relevant difference between the two Phases.

### Evidence

The assumptions of ANOVA were met as Shapiro–Wilk test for normality was not rejected, and Levene’s test for the equality of variances was not rejected [*F*_(__1_,_80__)_ = 1.416, *p* = 0.238 for Phase 1 and *F*_(__1_,_80__)_ = 0.942 *p* = 0.335 for Phase 2]. Sphericity was not performed as there are only two levels of the repeated measures factors.

Table 2 reports results for the ANOVA. There is a significant difference between Phase 1 and Phase 2 as *F* = 4.280, *p* = 0.042. There is no significant difference between MoL group and VM group as *F* = 1.347, *p* = 0.249. Very importantly, the interaction effect Phases × Groups is significant, *F* = 8.322, *p* = 0.005, indicating that memory decay – the significant difference between Phases 1 and Phase 2 – differs between MoL group and VM group. The covariate “age” is significant in its main effect *F* = 4.165, *p* = 0.045 between groups. No significant difference in terms of main effect and interaction was found for gender. With reference to Within Subjects, the effect size is small for Phases (ω^2^ = 0.024), Phases × Group (ω^2^ = 0.052), Phases × Age (ω^2^ = 0.014), with reference to Between Subjects, effect size is very small for Group (ω^2^ = 0.002) and small for Age (ω^2^ = 0.020). Hedges’g is 0.154.

**TABLE 2:** Results of two-factor ANOVA with repeated measures and covariates.

	Sum of squares	df	Mean square	*F*	*p*	ω ^2^
**Within subjects effects**						
Phases	81.577	1	81.577	4.280	0.042	0.024
Phases × Groups	158.627	1	158.627	8.322	0.005	0.052
Phases × age	56.655	1	56.655	2.972	0.089	0.014
Phases × gender	9.376	1	9.376	0.492	0.485	0.000
Residuals	1486.733	78	19.061			
**Between subjects effects**						
Groups	18.058	1	18.058	1.347	0.249	0.002
Age	55.847	1	55.847	4.165	0.045	0.020
Gender	3.633	1	3.633	0.271	0.604	0.000
Residuals	1045.807	78	13.408			

In order to articulate the results of the ANOVA, we conducted a *post hoc analysis* (with Bonferroni adjusted level) for pairwise comparisons among groups (MoL and VM) and repeated-measures (Phase 1 and Phase 2). Results are in Table 3.

**TABLE 3:** Results of *post hoc* analysis.

		Mean difference	SE	*t*	*p*_*bonf*_
***Post hoc* comparisons – groups × phases**					
MoL, Phase 1	VM, Phase 1	2.645	0.895	2.956	0.022
	MoL, Phase 2	3.775	1.011	3.734	0.002
	VM, Phase 2	2.465	0.923	2.671	0.050
VM, Phase 1	MoL, Phase 2	1.130	0.923	1.225	1.000
	VM, Phase 2	–0.180	0.979	–0.184	1.000
MoL, Phase 2	VM, Phase 2	–1.310	0.895	–1.465	0.871

In the MoL condition, memory decay between Phase 1 and Phase 2 is significant (*t* = 3.734, *p* = 0.002). In VM condition, memory decay between Phases 1 and Phases 2 is not significant. There is a significant difference between MoL and VM in Phase 1 (*t* = 2.956, *p* = 0.022), but there is no significant difference between MoL and VM in Phase 2. Furthermore, there is a significant difference between MoL in Phase 1 and VM in Phase 2 (*t* = 2.671, *p* = 0.050), but there is no significant difference between MoL in Phase 2 and VM in Phase 1.

#### Hypothesis 1

With reference to Hypothesis 1, in the short term, the traditional MoL was more effective than the VM, that is to say, subjects that used their individual-specific places (e.g., their house) as mnemonic tools exhibited a better recall, immediately after the task, with respect to the subjects that used the VM. Hence Hypothesis 1 is not rejected.

Individual-specific familiar places are probably more vivid and accessible with respect to a novel virtual environment, so they constitute a more reliable short-term-memory tool (cfr. Jamieson and Schimpf, 1980; Moè and De Beni, 2005). In the short-term the superior sense of immersion induced by virtual reality (which should help for better retention of information) is unable to compensate for the efficacy of the MoL, which results as a better, short-term mnemonic tool.

#### Hypothesis 2

With reference to Hypothesis 2, in the long term, there is no difference between the traditional MoL and the VM, that is to say, after only a week, the advantage of MoL disappears as subjects using the VM exhibit a performance similar to the subjects using the traditional MoL. Hence Hypothesis 2 is rejected.

Very importantly, the interaction effect indicates that memory decay (from Phase 1 to Phase 2) is significant in the MoL condition but not in the VM condition.

The potential reasons for hypothesizing a lower decay of memory in correspondence of the VM with respect to the MoL are plausibly related to the “cognitive availability” of familiar places, which work as a double-edged sword. Indeed, familiar places are probably as much vivid as much noisier because they are associated with many experiences in the subject’s everyday life. Precisely because familiar environments are cognitively available in the short term (they are part of a subject’s life, so as to be easily employed in the traditional MoL), they cannot be exclusively dedicated to memory processes in the long term. In the long term, the VM, allowing 3D immersive experience, exerts a powerful cognitive imprinting, more effective than just “thinking about” familiar places.

## Discussion

Our study shows that immediately after the task, the MoL is a mnemonic tool superior to the VM (Hypothesis 1); after 1 week, there is no difference between the VM and MoL (Hypothesis 2). Our study also shows that while the MoL presents a significant memory decay (after only 1 week), the same cannot be maintained for the VM, as there is no difference in recall between Phase 1 and Phase 2.

The comparison between MoL and VM should be understood not in absolute terms (which mnemonic tool is better) but in relative terms, i.e., whether the advantages of the VM are able to “challenge” the well-known MoL. Hence the fact that Hypothesis 2 is rejected (VM and MoL are equivalent) is, nevertheless, a relevant result for organizational applications: *precisely because VM and MoL present an equivalent performance in long-term recall task, VM should be preferable as it possesses a number of advantages, that are missing in the MoL:*

(1) Virtual memoryscapes are collective. While the traditional mnemonic strategies (i.e., MoL) are individual-specific, virtual memoryscapes represent a substantial innovation as they extend the peculiarities of individual navigation (typical of the MoL) to social groups (i.e., teams) so as to constitute a collective tool able to align long-term memory among the members of a social group. The possibility of creating cognitively shared contents probably represents the most relevant advantage of using virtual reality to enhance organizational memory.
(2) Virtual memoryscapes are controllable. An intrinsic limit of the traditional mnemonic strategies (such as MoL) is that they are not experimentally controllable: subjects exploit idiosyncratic resources (each subject uses her/his own house) as a memory tool. Hence a comparative assessment of memory performance is problematic because it is strongly dependent on individual-specific factors. VM is controllable as the same virtual environment applies to all the subjects. This guarantees that such a tool, when implemented in organizational settings, admits tailormade design and implementation able to meet the specific needs of a team.
(3) Virtual memoryscapes are dynamic. Being experimentally controllable, it is possible to constantly update the VM (but this is not possible for the traditional MoL). For example, it is possible to place new content or to update an existing one. In this way, it is possible to “update the collective memory” simply by instructing subjects to re-navigate the virtual environment in order to retain the new information. VM, being implemented through an HMD, can be navigated often and easily. Of note is that the efficacy of VM for retaining continuously updated information (requiring novel spatial navigation) has not been tested so far. The lack of evidence suggests exploiting this dynamic property parsimoniously and investigating it in future research.
(4) Virtual memoryscapes are non-manipulable. Information offloaded into external devices can be manipulated for deliberate purposes or erroneously (Risko et al., 2019). Virtual memoryscapes by enhancing cognitive memory are less subject to manipulation. If manipulated (i.e., some data are adulterated), the novel information generates a dissonance: adulterated data, present in the memoryscape, do not correspond to the ones retained in the cognitive memory of memoryscape users, so they are easily detected. Such dissonance could represent an effective control to erroneous adulteration and a deterrent to deliberate information manipulation.

Notice that a real memoryscape (not a virtual one), such as a real house or palace known by all the participants, could be used as a collective memory tool. *De facto*, it is possible to instruct subjects to navigate a common physical place to retain the specific contents located along the walking path. This strategy, though possible, is not easily implemented: it requires choosing an adequate physical place and asking participants to explore the place in controllable conditions. Virtual reality allows substituting physical places with tailormade virtual environments designed in order to fit specific organizational needs.

### Implications for Organizations

Memory is often a collective phenomenon, not bounded to individuals (Hirst and Manier, 2008; Hirst et al., 2018). For centuries, we are used to offloading memory by distributing information among the members of our social group. Each member of the social group not only remembers her/his own information but somehow knows what kind of information other members are storing. The distribution of information among the members of a social group is a central argument of transactive memory theory, which, after almost 40 years since its seminal contributions, represents a traditional topic of organizational literature (see Wegner et al., 1985; Wegner, 1987). In organizations, teams constitutively rely on distributed information: each member delegates others to remember specific information and is entrusted with remembering its own. Information offloaded onto team members is broader and richer if compared to the one that a single member handles. Such distributed information tends to bind the members and free their cognitive resources (Brandon and Hollingshead, 2004; Zhang et al., 2007; Peltokorpi, 2008). While in its original formulation, transactive memory theory placed emphasis on the fact the knowledge was distributed among the members of a social group (Wegner, 1987), in its recent reformulation, such members are somehow substituted by digital technologies that constitute a handy and dynamic transactive memory (Wegner and Ward, 2013). Generally speaking, information systems can be helpful to extend transactive memory to larger groups to create a transactive memory that is no longer defined in the context of small groups, but that is valid for the entire organization (Nevo and Wand, 2005).

The possibility of extending memory outside individual brain-bounded boundaries, is related to the notion of extended cognition: it relies on the hypothesis that a number of cognitive processes are made possible either through the use of internal resources (i.e., brain) or external artifacts (Clark and Chalmers, 1998). Part of our memory can be transferred to external resources with an evident advantage of reducing cognitive load. In this perspective, cognitive offloading is an adaptive strategy used to free cognitive resources (Sparrow and Chatman, 2013; Heersmink, 2016). For example, whenever we use a calendar to keep a record of future meetings, we are offloading information that otherwise should be retained in our cognitive memory. But offloading is also maladaptive, as there is evidence of a general decrease of memory performance properly because the internal memory is substituted by external devices (Carr, 2011; Greenfield, 2014), contributing to generalized digital amnesia (Sparrow et al., 2011; Wegner and Ward, 2013). Furthermore, offloading facilitates information manipulation (Risko et al., 2019).

Hence, the pillar of information systems (“everything should be offloaded”) could become the main cause of organizational digital amnesia: organizational actors are used to forgetting information precisely because they expect to rely on external memory devices, such as information systems. But such digital amnesia, which is, by definition, an individual phenomenon, presents systematic risks if its consequences spread in the whole organization. The maladaptive consequences of memory offloading (for an updated overview, see Heersmink and Sutton, 2020) are relevant in organizational settings, in which such maladaptive consequences could become systematic when they are not anymore idiosyncratic (limited to single subjects) but are “shared” among organizational members. The more organizational actors offload information, the less they possess at hand information to interact with other organizational members and to accomplish tasks. The less they possess at hand information, the more maladaptive consequences spread in the social group producing endemic consequences. For instance, the fine-grained content of a complex insurance contract (like the one we used in our experiment) can be surely managed through a dedicated information system, but this does not substitute that its content must be memorized (also in a coarse-grained manner) by decision-makers to be effectively used. If the decision-makers are not aligned – i.e., do not possess the same level of knowledge of the contract – they will be unable to use, on the fly, its content and effectively interact. Furthermore, they could make errors in modifying the contract. Due to their unaligned memory, they could create inconsistencies in the content of the contract, caused by their inability to handling its content as a “whole.”

From the normative point of view, we argue that virtual memoryscape can be used (i) to fix the maladaptive consequences of massive offloading so as to counterbalance organizational digital amnesia; (ii) to align memory of the members of social groups (i.e., teams), overcoming the limits of traditional mnemonic methods (such as MoL) that are individual-specific.

### Limitations and Future Research

Our study presents a number of limitations:

(1) In our study, we did not consider cybersickness symptomatology, such as nausea, dizziness, disorientation, fatigue, and instability, which stems from the implementation of VR systems (Kourtesis et al., 2019). Such symptomatology negatively affects cognitive and behavioral performance, and user experience. Despite the maturity of VR technology, how to evaluate this symptomatology is debated (see Somrak et al., 2021, for a comparison of cybersickness questionnaires).
(2) Researchers’ technological competency on HMD hardware and software are paramount to reduce adverse symptoms and effects of virtual reality application, so as to ensure the health and safety standards. Inappropriate headsets produce cybersickness symptomatology and negatively affect performance and experience. In our experiment, we used an Oculus Go but other devices (such as Oculus Quest), able to better mitigate such negative effects (Kourtesis et al., 2019), are available. Consider, also that, in our experiment, the limited exposure to the virtual environment (10 min) limits the inductions of adverse symptomatology.
(3) Our experiment is characterized by the absence of ergonomic interactions and haptic sensations, and this reduces immersion. Haptic and visual working memory are strictly related (Morimoto, 2020), and the integration of multisensory information is crucial for exploratory or recalling purposes (Sciutti et al., 2019). Our study does not consider such aspects.
(4) We cannot exclude that one factor that might affect performance in the VM condition is the novelty, and in particular the excitement to use a novel technology (HMD). Indeed, we cannot exclude that this factors made the participants in the VM more motivated and enthusiast, that is to say, excitement for novelty, and not just immersive experience, played a role.
(5) A fundamental feature of our design is the predefined association between place/object and memory in the virtual environment. This aspect normally constitutes a limitation in standard applications in which personalization is desirable. Actually, this feature is a specific methodological choice in our study. The possibility of creating a virtual environment that *applies to all the subjects* (without a personalization) is precisely the organizational application that we are testing in order to align collective memory in a controllable manner. The notion of memoryscape is, by definition, collective (as we discuss in section “Introduction”). In our study, we were not interested in memory performance in absolute terms but in relative terms: we compared the VM (which applies to a social group) with respect to the MoL (which is individual-specific).

Results of our study show that the virtual memoryscape is not superior to the MoL in the short term, but it is equivalent one week later. But there are two caveats:

1. In our experiment, memory decay (the difference of memory performances between Phase 1 and Phase 2) is related to a time window of a week. If we project the decay on a longer-term, consistently with Ebbinghaus’ forgetting curve (Ebbinghaus, 1880; Murre and Dros, 2015), we can reasonably assume that VM will surpass the traditional MoL. Put differently, there are reasons to hypothesize that memory performance based on MoL is probably less reliable in the long-term with respect to the one based on a VM.
2. In our virtual environment (treatment) we did not consider the role of ergonomic interactions and haptic sensations, and this represents a fundamental limitation (as discussed a few lines above). We cannot exclude that the introduction of ergonomic interactions and haptic sensations will increase the effect size in favor of the VM.

We postpone to future research the comparison between MoL and VM on longer-terms, taking into account of the limitations discussed above.

## Conclusion

Despite the maturity of virtual reality technology and the increasing application for memory purposes, implementations in organizational settings are lacking. Our study represents a first attempt as it was implemented in strict ecological conditions: (i) participants were recruited from large banking group in which they had responsibilities in their business unit, (ii) experimental task was very similar to real tasks they accomplish in standard working activities, (iii) spatial navigation occurred in virtual places consistent with experienced workplaces.

Our experiment was not addressed to assess memory performance in absolute terms but to evaluate the virtual memoryscape (which is a novel, collective tool and could be salient in organizational settings) with respect to the traditional individual-specific MoL, representing a benchmark. In many organizational activities (such as the cases of team members working on complex projects), there are types of information that are simply shared (and not distributed as it happens in transactive memory) as all the team members must be aligned to remember the same contents. And often, such contents create a significant cognitive load, are continuously updated and must be retained without errors. In such situations, virtual memoryscapes came in help as they represent mnemonic tools that are intrinsically collective, provide a solution to the relevant problem of *memory alignment of team members*, and are able to support the creation of shared cognitive memory.

Our emphasis on memory enhancement through virtual memoryscapes is not simply motivated by the necessity of “fixing” digital amnesia and “counterbalancing” massive offloading in organizations. There is more. In the last two decades, the explosion of ICT has led to an increase of complexity, as technological advancements had an impact on connectivity among people and devices, computational power and storage of information (Merali, 2004; Hanseth and Lyytinen, 2016). The advanced use of information systems became a mantra, not just for strictly functional and operational reasons but also because information systems represent critical resources with a strategic potential (Pearlson et al., 2019). Such an explosion of information often translates to increasing complexity with a great impact on existing business models. Far from producing only digital amnesia, information systems often generate complexification, which must be managed to require, to a greater extent, the use of dedicated cognitive resources.

Concluding, the use of virtual memoryscapes should be conceptualized as a novel digital tool that meets the challenges faced by many organizations in the age of complexity. Decision-makers are, more and more, involved in collective complex projects that require effortful cognitive processes characterized by significant cognitive burden. Digital technologies could come in help by augmenting cognitive faculties.

## Data Availability Statement

The datasets presented in this article are not readily available. Requests to access the datasets should be directed to corresponding author (a.mastrogiorgio@imtlucca.it).

## Ethics Statement

The studies involving human participants were reviewed and approved by the Area Vasta Nord Ovest Ethics Committee (protocol n. 24579/2018). The patients/participants provided their written informed consent to participate in this study.

## Author Contributions

AM: validation, formal analysis, writing – original draft, writing – review & editing, and visualization. FZ: methodology and investigation. FM: project administration and funding acquisition. ER and NL: writing – review & editing, and funding acquisition. APM: conceptualization, methodology, writing – review & editing, and supervision. All authors contributed to the article and approved the submitted version.

## Conflict of Interest

FM was employed by Intesa Sanpaolo Innovation Center S.p.A. The remaining authors declare that the research was conducted in the absence of any commercial or financial relationships that could be construed as a potential conflict of interest.

## Publisher’s Note

All claims expressed in this article are solely those of the authors and do not necessarily represent those of their affiliated organizations, or those of the publisher, the editors and the reviewers. Any product that may be evaluated in this article, or claim that may be made by its manufacturer, is not guaranteed or endorsed by the publisher.
